# Delirium is prevalent in older hospital inpatients and associated with adverse outcomes: results of a prospective multi-centre study on World Delirium Awareness Day

**DOI:** 10.1186/s12916-019-1458-7

**Published:** 2019-12-14

**Authors:** Geriatric Medicine Research Collaborative, Carly Welch, Lauren McCluskey, Daisy Wilson, George E. Chapman, Thomas A. Jackson, Jonathan Treml, Daniel Davis, Emma Cunningham, Claire Copeland, Terrence Quinn, Thomas Pinkney, Rahul Mahida, Peter Nightingale, Sarah Richardson, Oliver Todd, Ruth Willott, Kelli Torsney, Mary Ni Lochlainn, Kumudhini Giridharan, Natalie Cox, Jane Masoli, Lindsay Ronan, Victoria Gaunt, Benjamin Jelley, Joanne Taylor, Roisin Healy, Emily Rose, Megan Parkinson, Ajay Macharouthu, Eilidh McKenzie, Roisin McCormack, Jasmine Hart, Alison McCulloch, Neil Henderson, Louise Beveridge, Emma Elliott, Bogna Drozdowska, Martin Taylor-Rowan, Natasha Christodoulides, James Allen, Harriet Brown, Jennifer Champion, Riana Patel, Ghazal Hodhody, Kara Mayor, Christopher James Miller, Mark Studley, Vishnu Prasad, Emma Mumtaz, Sam Cohen, Sherif Abdelbadiee, Anna Lewis, Bushra Khizar, Hannah Pendleton, Teresa Harkin, Steve Rutter, Ayoub Behbahani, Abolfazl Behbahani, Ani Tencheva, Rachel King, Laura Jones, Alex Hornsby, Robbie Horton, Kate Foster, Kirsty Moore, Vincent McCormack, Matthew Kearney, Emma Fisken, Rory Durcan, Elizabeth Deacon, Jane Noble, Arunkumar Annamalai, Roxana Taranu, Michael Sen, Pryankaran Mithrakumar, Laura Briggs, Jamal Bhatti, Shiv Bhakta, Amaka Achara, Elizabeth Ellis, Sejlo Koshedo, Ayesha Aamir, Edward Wu, Abdullah B. Khalid, Parrthiepan Visvaratnam, Ijeoma Obi, Nader Nashed, Chioma Iwu, Sneha Gurung, Shonit Nagumantry, Olugbenro Akintade, Valerie Page, Kwasi Debrah, Katie Ball, Jabed Ahmed, Zhao Xiao Bei, Sarah B. McClelland, Michael Haley, Norman Pang, Andre Le Poideven, Emily Moore, Freya Cooper, Natalie Grundmann, Elizabeth Lonsdale-Eccles, Janine Valentine, Emma Stratton, Emily Bowen, Miriam Thake, Dorothy Kuek, Wioletta Pyc, Deborah Scott, Frances Rickard, Natalie Gaskell, Helen McDonald, Victoria Gaunt, Sam Mills, Stuart Winearls, Paapa A-Odame, Ciaran Barlow, Isabelle Nicholls, Emma Norman, Kim Kirrane, Peter Jackson, Christian Chourot, Laura Jayne Beeley, Aaron Kay, Victoria Clayton, John Marshall, Hannah Morgan, George Naish, Clare Hunt, Rajeev Mishra, Saurav Bhattacharya, Nisha Rai, Ahmad Alareed, Clementine Anderson, Ganapathy Bhat, Sandra Darko, Pedro Vila De Mucha, David Saliu, Karen Beharry, Laurence Caines, Sanojan Bremakumar, Daniel Furmedge, Celine Bultynck, Esther Hindley, Elaine Seymour, Darmiga Thayabaran, Cal Doherty, John Frewen, Oluwatosin O. Abiola, Simon Tetlow, Guy Tinson, Olivia Morrow, Isabel Greaves, Rachael Bygate, Aayenah Yunus, Catherine Bryant, Howell Jones, Helen Bowden, Rose Laud, Keziah Austin, Farrah Bahsoon, Martin Glasser, Khai Lee Cheah, James Speed, Lucy Porter, James Dove, Katrin Hoffman, Olivia Evans, Taran Nandra, Leeying Giet, Simon Stapley, Imola Bargaoanu, Ismail Kadir, Adam McClean, Pranav Mishra, Katie Houldershaw, Ana Andrusca, Emmy Abu, Adam Swietoslawski, Bilquis Ahmed, Matthew Ansell, Saad Abdullah, Shoaib Iqbal, James Wilcockson, Angela Kabia, Karthika Velusamy, Nihaad Syed, Charlotte Chuter, Hamza Ahmed, Sarah Ahmad, Gladys Ofoche, Jacqueline Ibanichuka, Alice Wheeler, Angharad Chilton, Zainab Hussain, Felicia Tan, Sinead Quinn, Paul Croft, Amy Walker, Charlotte Bell, Claire Wilkes, Eliza Griffiths, James Reid, Ahmed Abras, Muhammad Adam, Awolkhier Mohammedseid-Nurhussien, Sohail Shakeel, Zarah Amin, Georgia R. Layton, Nathan Ingamells, Jemima Taylor, Luke Wynne, Wan Idoracaera Calisa Ikhwan, Hanna Waraich, Olivia Cooper, Philip Thomas, Emily Williamson, Huma Naqvi, Helena Lee, Elizabeth Holmes, Megan Offer, Alex McQuillan, Emma Jay, Hannah Currie, Sureena Janagal, Gary Kumbun, Rodric Jenkin, Holly Jacques, James Gaywood, Laura Babb, Moe Su Su San, Sasha Porter-Bent, Daisy Wilson, Tarunya Vedutla, Asiodu Nneamaka, Anum Cheema, Hannah Moorey, Asma Khan, Zeinab Majid, Puja Jatti, Abhishek Gupta, Tammy Lee, Helen Chamberlain, Clare Hughes, Alexis Giles, Tamsin Critchlow, Bethan Morgan, Alice Moseley, Grace Fennelly, Sophie Pettler, Edward Bilton, Emma Astaire, William McKeown, Katherine Williamson, Caroline Rice, Sharan Ramakrishna, Zahid Subhan, Nedaa Haddad, Anjli Krishan

**Affiliations:** Institute of Inflammation and Ageing, University of Birmingham, Edgbaston, B152TT UK

**Keywords:** Delirium, Frailty, Older adults, Collaboration

## Abstract

**Background:**

Delirium is a common severe neuropsychiatric condition secondary to physical illness, which predominantly affects older adults in hospital. Prior to this study, the UK point prevalence of delirium was unknown. We set out to ascertain the point prevalence of delirium across UK hospitals and how this relates to adverse outcomes.

**Methods:**

We conducted a prospective observational study across 45 UK acute care hospitals. Older adults aged 65 years and older were screened and assessed for evidence of delirium on World Delirium Awareness Day (14th March 2018). We included patients admitted within the previous 48 h, excluding critical care admissions.

**Results:**

The point prevalence of Diagnostic and Statistical Manual on Mental Disorders, Fifth Edition (DSM-5) delirium diagnosis was 14.7% (222/1507). Delirium presence was associated with higher Clinical Frailty Scale (CFS): CFS 4–6 (frail) (OR 4.80, CI 2.63–8.74), 7–9 (very frail) (OR 9.33, CI 4.79–18.17), compared to 1–3 (fit). However, higher CFS was associated with reduced delirium recognition (7–9 compared to 1–3; OR 0.16, CI 0.04–0.77). In multivariable analyses, delirium was associated with increased length of stay (+ 3.45 days, CI 1.75–5.07) and increased mortality (OR 2.43, CI 1.44–4.09) at 1 month. Screening for delirium was associated with an increased chance of recognition (OR 5.47, CI 2.67–11.21).

**Conclusions:**

Delirium is prevalent in older adults in UK hospitals but remains under-recognised. Frailty is strongly associated with the development of delirium, but delirium is less likely to be recognised in frail patients. The presence of delirium is associated with increased mortality and length of stay at one month. A national programme to increase screening has the potential to improve recognition.

## Background

Delirium is a neuropsychiatric syndrome, which disproportionately affects older people in hospital. The Diagnostic and Statistical Manual on Mental Disorders, Fifth Edition (DSM-5) defines delirium as an acute and/or fluctuating change in awareness, arousal, and other cognitive deficits due to physical illness or drugs [1, 2]. Psychomotor subtypes are hyperactive, characterised by motor agitation, perceptual differences, and delusions, and hypoactive, featuring predominantly motor retardation and thought process abnormality; or mixed [3]. It is very common, but prevalence differs across populations: 10–31% for most acute settings outside critical care [4]. Prior to our study, the largest point prevalence study of delirium using DSM criteria reported a prevalence of 19.6% amongst 280 general hospital adult inpatients in a single centre in Ireland [5].

Delirium is consistently associated with increased mortality, accounting for age, co-morbidity, and acute illness [6]. It is also associated with increased length of hospital stay, new institutionalisation, and distress to patients and families [4, 7]. Delirium commonly occurs in people with dementia [8] and is considered to worsen cognitive decline [9]. In people without dementia, an episode of delirium is associated with eightfold increased risk of later-life dementia diagnosis [10]. Few studies have assessed the relationship between delirium and frailty [11–14].

Delirium remains underdiagnosed in up to three quarters of patients [15–18]. Incomplete understanding of delirium and resultant educational needs of healthcare professionals, alongside avoidant behaviours towards a challenging patient group, are likely contributory [19]. National Institute for Health and Care Excellence (NICE) Guidelines recommend that all patients aged 65 or over are screened for delirium upon hospital admission [20]; this can be done using the 4 A’s Test (4AT). The 4AT is a validated screening tool, which can be completed by any healthcare professional in less than 2 min [21]. Diagnosis should be made using DSM-5, recorded in inpatient notes, and communicated to the general practitioner [22].

This study set out to identify the point prevalence of delirium, rates of screening, and rates of recognition of delirium in non-elective admissions of older people within the UK. We aimed to assess patient and hospital factors inclusive of frailty measures that were predictive of delirium, screening, and recognition. Secondary outcomes included one-month mortality and length of stay.

## Methods

### Study design and setting

We conducted a multi-centre study of delirium screening, recognition, and discharge documentation on Wednesday 14th March 2018: World Delirium Awareness Day. This is an annual international day, which aims to increase awareness of delirium amongst healthcare professionals, patients, carers, and stakeholders [23]. This day was chosen to encourage an increased atmosphere acknowledging the importance of delirium screening and recognition within participating trusts and to encourage quality improvement strategies for the future. This involved acute care trusts within the UK who volunteered to participate. Participation was open to all hospitals within the UK, and the onus was put upon staff within individual sites to volunteer to participant rather than being selected. No financial incentives were provided to trusts to participate. Regional representatives emailed the staff at all sites to encourage participation. Data collected from each site were anonymised and entered into pre-formatted Excel spreadsheets. Spreadsheets were formatted so that data was entered in the same way for all sites. These spreadsheets were collated centrally. Our reporting is in line with Strengthening the Reporting of Observational Studies in Epidemiology (STROBE) guidelines (Additional file 3) [24].

### Participants

Inclusion criteria were adults aged ≥ 65 years (in line with NICE guidelines), admitted between 08:00 12th March 2018 and 07:59 14th March 2018, and still hospital inpatients at the time of assessment. Exclusion criteria were patients admitted to critical care, imminently approaching the end of life, or in whom it was impractical to assess for logistical reasons (e.g. patient undergoing operation, not at bedside). The minimum data required for inclusion was 4AT score and presence/absence of delirium.

### Delirium screening and assessment

Patient assessment as part of the study took place between 08:00 and 20:00 on 14 March 2018. All included patients were screened using the 4AT by the study team; in all patients who scored ≥ 4/12, a further assessment for delirium was conducted. Our approach to further assessment for delirium was operationalised on DSM-5 (Additional file 1). The 4AT was performed by a healthcare professional or student with training and support from the local geriatric medicine site lead. All formal DSM-5 assessments were performed by a healthcare professional; patients with 4AT score ≥ 4/12 identified by students were reviewed by healthcare professionals. Standardised training was provided centrally to all via webcast and video resources. The presence or absence of delirium was classified as definite (meets DSM-5 criteria), possible (meets some DSM-5 criteria but not all), or no delirium. Motor subtype was classified by the Delirium Motor Subtype Scale (DMSS) [19] as hypoactive (reduced alertness), hyperactive (increased alertness or motor agitation), mixed (some features of both hypoactive and hyperactive), or no clear motor subtype.

### Additional data collection

Further patient details were recorded from the patient’s hospital notes including age, gender, dementia status (known history—any history documented in the notes/probable—no documented diagnosis but history of progressive cognitive impairment impairing activities of daily living documented/no dementia), and specialty at time of assessment (acute medicine/geriatric medicine/stroke/other medicine/orthopaedic surgery/general surgery/other surgery). Clinical Frailty Scale (CFS) from 1 to 9 was determined prospectively from note review (functional status documentation) and clinical assessment by the student or healthcare professional assessing the patient as part of the study [25]. We recorded if patients had been screened for delirium (using any recognised tool) by the usual care team and if a diagnosis of delirium was recorded in the medical notes by the usual care team prior to 4AT assessment as part of this study. Delirium was considered to have been recognised if a DSM-5 diagnosis was made during the assessment, and this had previously been documented by the usual care team. Each site collected data on local factors: presence of local delirium guidelines, local delirium patient leaflets, delirium screening tools in admission documentation booklets, geriatric medicine team embedded into the admissions unit, or a specialist delirium team. These were defined locally. Length of stay, mortality, and documentation of delirium on discharge documentation were collected up until 13th April 2018.

### Statistical methods

Statistical analysis was performed using IBM SPSS Statistics 22 (Chicago, IL, USA). We assessed the differences between patients with and without delirium using chi-squared tests for categorical data and independent *t* tests for continuous data. Grouping of variables was decided post hoc. Possible delirium was coded as no delirium, and probable dementia was coded as dementia. Frailty status was separated into three categories by CFS (fit, 1–3; frail, 4–6; very frail, 7–9). Delirium subtype was classified as hypoactive or other. Due to small numbers, general and other surgery specialties were grouped for the main analysis. General and other surgery were further grouped with orthopaedic surgery for recognition analysis; stroke was grouped with other medicine when analysing discharge documentation.

Binary logistic regression was performed to assess the effect of covariates upon delirium screening, prevalence, and recognition, and the effect of screening upon recognition. Patients who died within the follow-up period were excluded from the length of stay analysis. Length of stay was visually assessed for normality; Mann-Whitney *U* test was used to assess the length of stay in those with and without delirium. Effects of the presence of delirium upon length of stay were assessed using robust (bootstrapped) analysis of covariance (ANCOVA), adjusting for the variables above. Bootstrapping was performed due to skewed distribution of length of stay using IBM SPSS Statistics 22, with the number of samples set at 1507, confidence interval set at 95%, and a simple sampling method selected. Bootstrapping was not performed for analysis of other outcomes. Association of delirium with mortality was assessed using binary logistic regression and Cox regression. A secondary analysis was performed to assess the effects of recognition and delirium subtype upon length of stay and mortality. Any missing variables and outcome data were coded as missing data, but these participants were included in all analysis, provided data was available on the presence or absence of delirium.

### Ethical approval

All data were collected as part of a multi-centre audit to assess compliance with NICE guidelines and registered through clinical governance departments. Anonymised data were securely transferred to the University of Birmingham. Ethical approval was obtained for a secondary analysis of the anonymised database from the University of Birmingham Science, Technology, Engineering, and Mathematics Ethical Review Committee (ERN_18-1415).

## Results

**Fig. 1:**
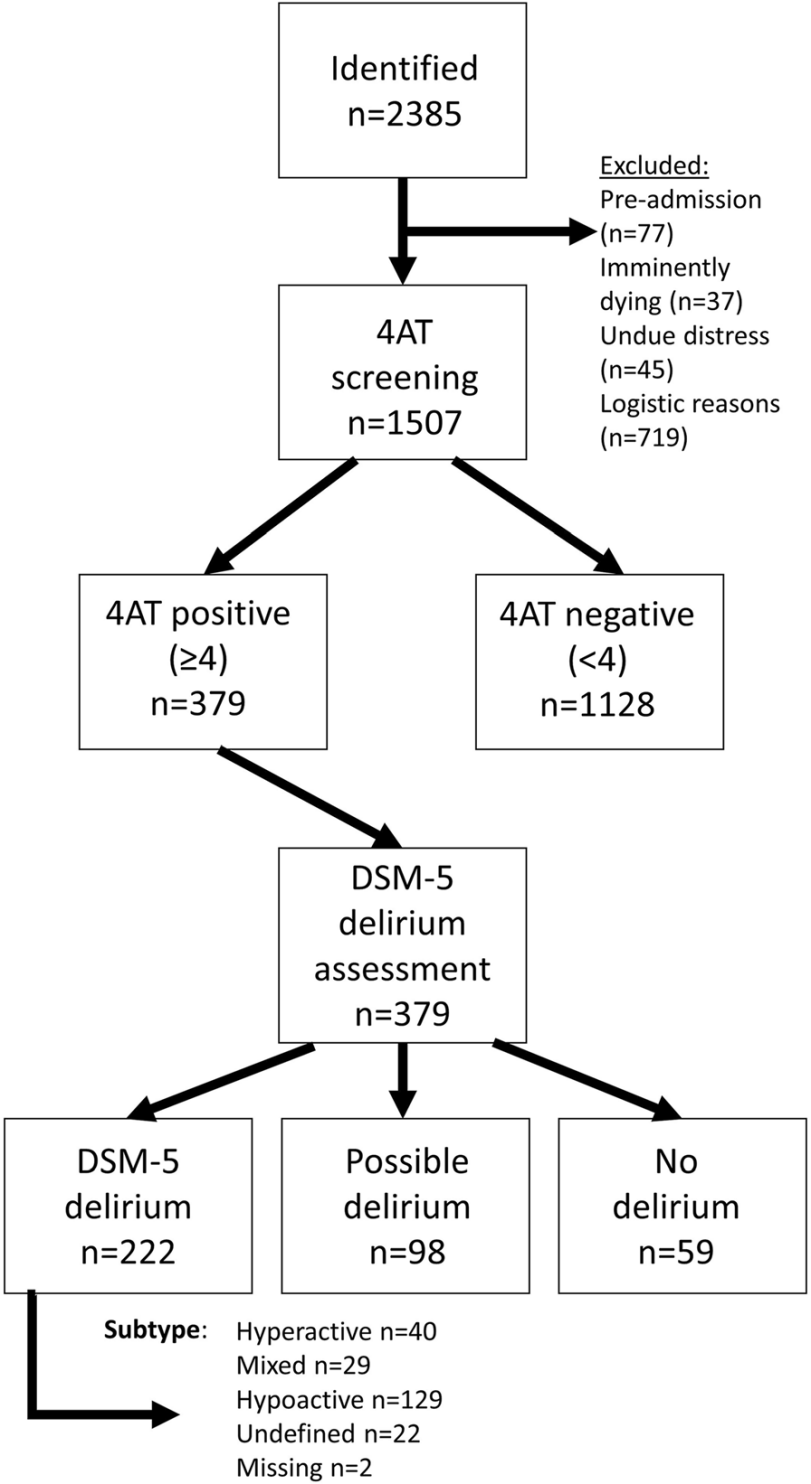
Flow chart demonstrating participation in delirium day study. On 14th March 2018, 45 hospitals participated in World Delirium Day study. Two thousand three hundred and eighty-five individuals met the study criteria of admission between 08:00 on 12th March 2018 and 07:59 on 14th March 2018. Thirty-seven individuals were excluded as they were judged to be imminently dying. Seven hundred and nineteen individuals were excluded for logistical reasons. Seventy-seven were excluded as they had not yet had their initial assessment. Forty-five were excluded because the assessment was deemed to cause undue distress. One thousand five hundred and seven individuals were screened with 4AT. Of these, 366 had a score equal to or greater than four and underwent further assessment of delirium using DSM-5 criteria. Of those who were 4AT positive, 222 were proven to have DSM-5 delirium

A total of 45 hospitals participated (Additional file 2: Figure S1), and 2385 patients were identified. Reasons for exclusion included the following: pre-admission (77), imminently dying (37), undue distress (45), and logistical reasons (719). Logistical reasons included the patient or notes being unavailable, or unavailability of staff. The final sample included 1507 patients (Fig. 1). The mean age was 80.0 (SD ± 8.3); 54.2% were female, 16.3% had dementia or probable dementia, 43.0% were acute medicine patients, and 68.1% had a CFS score of 4 or greater (Table 1; Additional file 2: Table S1). Mortality to follow-up was 6.7% (97/1507). The rates of missing data were low overall; the rates of missing data are included within Additional file 2: Table S2.

**Table 1:** Demographics of patients included in this study. Results are shown for the percentage of study participants who met each characteristic. These have been further separated for comparison between participants with and without delirium. Overall, the mean age of participants was 80.0; 54.2% were female, and 16.3% had known or probable dementia; 43.0% were admitted under acute medicine at the time of assessment; 68.0% had a CFS score of 4 or greater

	All	No delirium	Delirium (DSM-5)	*p*
Age (mean, SD)	80.0 (8.3)	79.3 (8.3)	84.0 (7.4)	< 0.001
Gender				
Female	54.2% (798)	52.9% (663)	62.0% (218)	0.013
Dementia				
Known or probable	16.3% (244)	13.0% (166)	35.5% (78)	< 0.001
Specialty				
Acute medicine	43.0% (648)	42.2% (542)	47.8% (106)	< 0.001
Geriatric medicine	17.6% (265)	16.0% (206)	26.6% (59)	
Other medicine	20.9% (315)	22.1% (284)	14.0% (31)	
Stroke	3.7% (56)	4.0% (52)	1.8% (4)	
General and other surgery	8.5% (128)	9.4% (121)	3.2% (7)	
Orthopaedic surgery	6.3% (95)	6.2% (80)	6.8% (15)	
Frailty				
Fit (CFS 1–3)	31.9% (468)	36.3% (453)	6.9% (15)	< 0.001
Frail (CFS 4–6)	54.3% (796)	53.0% (662)	62.0% (134)	
Very frail (CFS 7–9)	13.7% (201)	10.7% (134)	31.0% (67)	

### Delirium prevalence

**Fig. 2:**
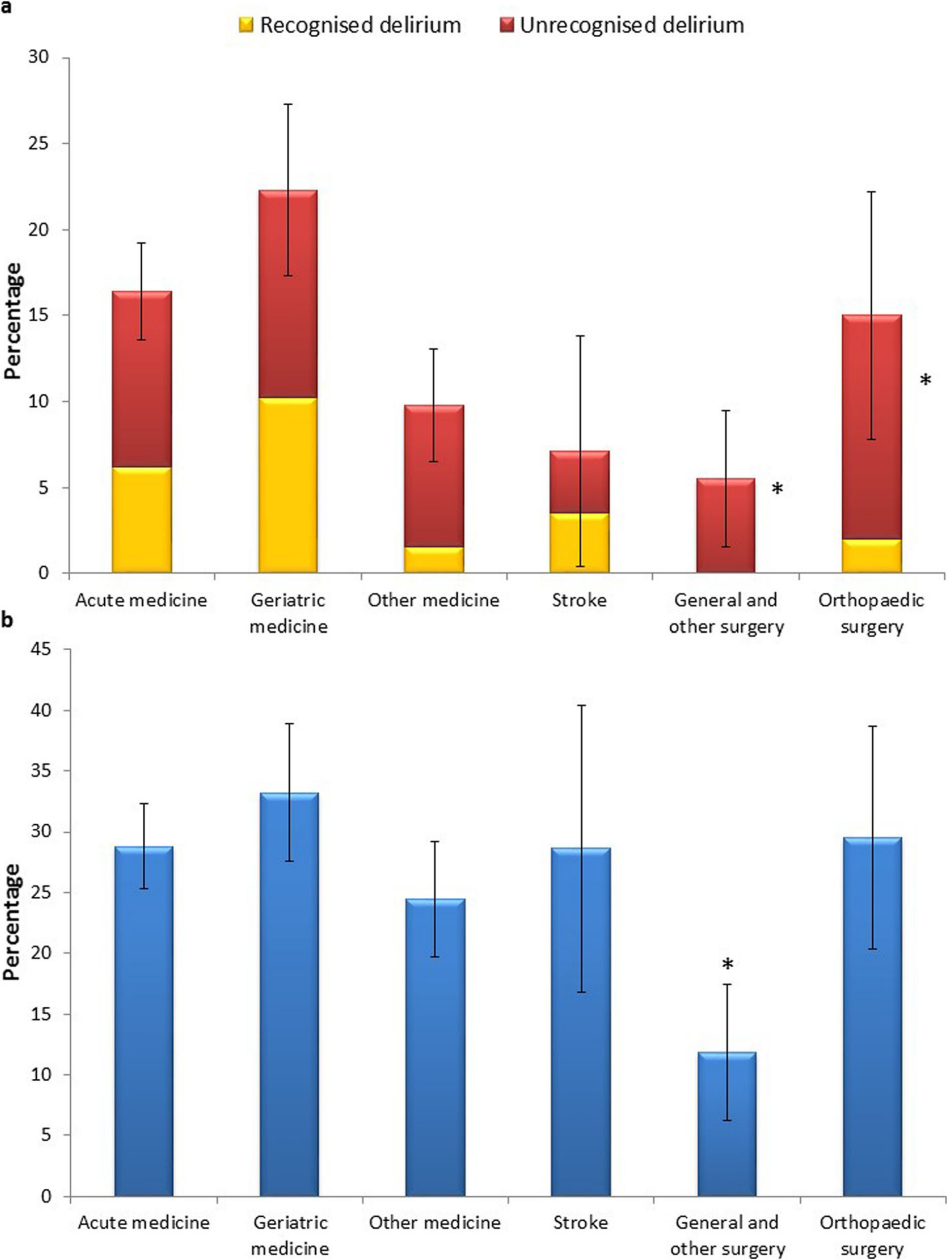
**a** Prevalence of recognised and unrecognised delirium by specialty. The total of each bar represents the overall prevalence of delirium within each specialty; standard error bars show the 95% confidence intervals of prevalence by specialty. The yellow portion of each bar represents recognised delirium, and the red portion of each bar represents unrecognised delirium. Prevalence differed between specialties; however, after controlling for other confounders (e.g. age), specialty was not predictive of delirium prevalence. There were reduced odds of recognition of delirium in patients admitted to general, other, or orthopaedic surgery as compared to acute medicine. **b** Screening of delirium by specialty. Each bar represents the total percentage of patients who were screened for delirium by the usual care team prior to assessment as part of this study within each specialty; the standard error bars show the 95% confidence intervals of percentage screened. Reduced odds of screening for delirium were exhibited in patients admitted under general or other surgery as compared to acute medicine

With 4AT assessment, 25.1% (379/1507) scored positive (≥ 4/12). Prevalence of DSM-5 delirium was 14.7% (222/1507); including those with possible delirium as well as DSM-5 delirium, prevalence was 21.2% (320/1507). Considering delirium subtypes, 18.2% (40/220) were hyperactive, 13.2% (29/220) were mixed type, 58.6% (129/220) were hypoactive, and 10.0% (22/220) had no clear subtype (as assessed by local data collectors). The presence of delirium was independently associated with increased age (per year of life: OR 1.04, CI 1.02–1.06; *p* < 0.001), dementia status (OR 1.95, CI 1.36–2.79; *p* < 0.001), frailty, frail (OR 4.80, CI 2.63–8.74; *p* < 0.001), and very frail (OR 9.33, CI 4.79–18.17; *p* < 0.001). Delirium prevalence was not affected by any hospital factors or gender (Additional file 2: Table S2). Prevalence of delirium differed between specialties (Fig. 2a). However, specialty did not affect the likelihood of delirium after adjusting for age and frailty (Additional file 2: Table S3).

### Delirium screening

There was evidence of delirium screening by the usual care team in 27.3% (410/1507). Increasing age (per year of life: OR 1.04, CI 1.02–1.06; *p* < 0.001) and the presence of a local delirium specialist team (OR 2.03, CI 1.48–2.80; *p* < 0.001) were associated with an increased chance of screening. Admission under general or other surgery compared to acute medicine resulted in a reduced chance of delirium screening (OR 0.38, CI 0.21–0.70; *p* = 0.002) (Fig. 2b). Chances of delirium screening by the usual care team were not affected by gender, dementia status, frailty, or other hospital factors (Additional file 2: Table S4).

### Delirium recognition

The usual care team recognised DSM-5 delirium in 34.2% of cases (76/222). Increased screening rates were associated with increased recognition rates by the usual care team (OR 5.47, CI 2.67–11.21; *p* < 0.001). The presence of a delirium team was associated with a decreased chance of recognition (OR 0.33, CI 0.23–0.84; *p* = 0.020). Delirium was less likely to be recognised in very frail compared to fit patients (OR 0.16, CI 0.04–0.77; *p* = 0.021) and general, other, and orthopaedic surgery patients compared to acute medicine (OR 0.04, CI 0.01–0.36; *p* = 0.004) (Fig. 2a). Age, gender, and dementia status did not impact upon delirium recognition. Delirium recognition was not affected by delirium subtype (Additional file 2: Table S5).

### Discharge documentation

Discharge documentation was assessed in 69.4% (154/222) with DSM-5 delirium. Delirium was documented on discharge summaries in 28.6% (44/154) of these. Documentation on discharge summaries was not associated with any hospital or patient factors (Additional file 2: Table S6).

### Delirium and length of stay

The median length of stay in patients with DSM-5 delirium was 11 days (IQR 5–21), compared to 7 days (IQR 3–14) in those without delirium (*p* < 0.001). This difference remained in multivariable analysis; delirium presence was associated with a mean (bootstrapped) increased length of stay of + 3.45 days (CI 1.75–5.07) compared to those without (*p* = 0.001). Possible delirium was associated with increased length of stay when compared to patients without delirium (+ 2.21 days, CI 0.27–4.52; *p* = 0.038); 4AT-positive status with no evidence of delirium was not associated with increased length of stay (Table 2; Additional file 2: Tables S7-S8). Further post hoc tests are included in Additional file 2: Tables S9-S11. There was no association of delirium recognition or subtype with length of stay (Additional file 2: Tables S12 and S13).

**Table 2:** Results of post hoc tests of mean difference following robust (bootstrapped) ANCOVA. The presence of delirium was associated with an increased length of stay of 3.45 days. Considering possible delirium separately, there was an increased length of stay of 2.21 days compared to those without delirium. Results of statistical significance (*p*<0.005) have been highlighted in bold

Status (*a*)	Status (*b*)	Mean difference (*a* − *b*)	Bootstrap				
			Bias	SE	*p*	95% confidence interval	
						Lower	Upper
No or possible delirium	Delirium	**− 3.45**	**0.017**	**0.84**	**0.001**	**− 5.07**	**− 1.75**
No delirium	4AT positive, no delirium	− 2.55	− 0.01	1.35	0.052	− 5.21	0.04
	Possible delirium	**− 2.21**	**− 0.07**	**1.08**	**0.038**	**− 4.52**	**− 0.27**
	Delirium	**− 3.95**	**− 0.02**	**0.91**	**0.001**	**− 5.78**	**− 2.22**
4AT positive, no delirium	Possible delirium	0.34	− 0.06	1.65	0.820	− 3.00	3.61
	Delirium	− 1.41	− 0.01	1.50	0.35	− 4.35	1.69
Possible delirium	Delirium	− 1.74	0.05	1.31	0.183	− 4.19	0.89

### Delirium and mortality

The presence of DSM-5 delirium was associated with increased mortality at 1 month both before (OR 3.02, CI 1.88–4.87; *p* < 0.001) and after adjusting for other variables: age, gender, frailty, specialty, and dementia (OR 2.43, CI 1.44–4.09; *p* = 0.001) (Table 3). Delirium was also associated with increased mortality in time-to-event analysis (HR 1.62, CI 1.00–2.61; *p* = 0.048) (Table 4). Possible delirium was associated with increased odds of death in univariable but not multivariable analysis (Additional file 2: Table S14). Similarly, 4AT-positive status was associated with increased odds of death in multivariable analysis (OR 2.55, CI 1.53–4.24; *p* < 0.001) (Additional file 2: Table S15). There was no effect of delirium recognition or subtype upon mortality (Additional file 2: Tables S16 and S17).

**Table 3:** Effect of delirium status upon odds of mortality within 30 days. Delirium was associated with an increased odds of death within 30 days both unadjusted and adjusted for other confounders. Of those other confounders measured, only being very frail (CFS 7–9) was associated with increased odds to death to 30 days. Results of statistical significance (*p*<0.005) have been denoted with *

	Coefficient	SE	Wald	Freedom	*p*	OR	95% confidence interval for OR	
							Lower	Upper
Delirium (unadjusted)	1.10	0.24	20.62	1	< 0.001*	3.02	1.88	4.87
Delirium (adjusted)^†^	0.89	0.27	11.02	1	0.001*	2.43	1.44	4.09
Very frail (adjusted)^‡^	0.95	0.38	6.20	1	0.013*	2.59	1.23	5.48

^†^Adjusted for age, gender, CFS, dementia status, and specialty

^‡^Adjusted for delirium status, age, dementia status, and specialty

**Table 4:** Effect of delirium status upon time to death with follow-up to 30 days. The presence of delirium was associated with a greater risk of an earlier death; no other variables were significant in time to death analysis

	Coefficient	SE	Wald	Freedom	*p*	HR	95% confidence interval for OR	
							Lower	Upper
Delirium (unadjusted)	0.58	0.23	6.25	1	0.012	1.78	1.13	2.81
Delirium (adjusted)^†^	0.48	0.24	3.90	1	0.048	1.62	1.00	2.61

^†^Adjusted for age, gender, CFS, dementia status, and specialty

## Discussion

In this multi-centre study of delirium in older acute hospital admissions, delirium was prevalent and associated with significant adverse outcomes. Delirium was more common in individuals with dementia and frailty. Delirium screening and recognition were both low. Importantly, higher screen rates were associated with fivefold higher recognition rates, demonstrating the need for screening in clinical practice. Delirium was associated with increased mortality and length of stay within one month of admission; this association remained after adjusting for age and frailty.

### Results in the context of other literature

Prevalence of delirium was lower than a previous single-site point prevalence study (14.7% vs. 19.6%) [5]. However, our study focussed on new admissions only; hospital-wide point prevalence may be higher including cases of incident delirium. Positive 4AT status prevalence was similar to a multihospital study [26]. Increased delirium prevalence was associated with age and dementia, as previously described [27, 28]. Prevalence of different delirium subtypes was similar to results published elsewhere [29].

Screening and recognition rates were similar to previous results [4, 18]. This is the first study to identify differences in delirium screening and recognition rates across specialties. Specialty was not predictive of delirium presence after adjusting for age, frailty, and dementia; specialty alone does not affect chances of delirium. However, individuals admitted under surgery were 3 times less likely to be screened for delirium and 20 times less likely to be recognised as having delirium. This may be related to the training and skill set of responsible healthcare staff.

Documentation of delirium on discharge summaries was poor. This was not affected by any hospital or patient factors. However, this effect is not unique to delirium; a similar rate of poor communication has been described across multiple settings [30]. A multitude of quality improvement projects has suggested techniques to improve discharge communication with varying success [31–33].

Small studies have demonstrated that frailty increases the risk of delirium [34–37] and is associated with greater mortality in delirium [37]. This is the largest study to examine this association. Patients with severe frailty were nine times more likely to have delirium, and delirium and frailty were independent risks of mortality and increased length of stay. The CFS is a widely used and valid method of measuring frailty in clinical practice [25], which can be used with minimal training by non-specialists [38]. We have uniquely demonstrated that frail patients were far less likely to have delirium recognised. This may be due to increased misdiagnosis as chronic cognitive impairment and a perception that features are “expected” for patients with frailty in hospital. By contrast, healthcare professionals may have a different subconscious bias in how they “expect” fit patients to present in hospital. Although we included cases of probable dementia as well as known dementia, it is likely that there were other patients who had undiagnosed dementia and additional patients with mild cognitive impairment; one fifth of patients with delirium have been shown to have undiagnosed dementia [39]. Frailty and cognitive impairment commonly co-exist [40]; therefore, some of the effects of reduced recognition of delirium may relate to pre-existent cognitive impairment.

### What is the internal validity of our research?

There are a number of important limitations. Firstly, usual clinical teams were informed of screening and diagnosis results; this may have moderated effects of non-recognition on outcomes. However, only a single study to date has found an association between non-recognition and adverse outcomes [41]. Secondly, non-specialists (healthcare staff other than geriatricians or psychiatrists) carried out the assessments. However, standardised training was provided to assessors, a structured proforma was used for assessment, and positive results were discussed with a local expert. In fact, delirium prevalence was slightly lower than previously published, suggesting if anything this method led to a higher specificity (i.e. fewer false positives). This in itself can be considered a limitation, although we purposefully used a strict interpretation of the DSM-5 criteria. The specificity of the 4AT against DSM-4 has previously been reported at 84.7%, whereas only 58.6% of 4AT-positive patients were considered to have DSM-5 delirium [21]. Thirdly, delirium tends to fluctuate, and delirium may not have been present when reviewed by the usual care team. The nature of delirium ascertainment was such that we may have missed delirium in those who were 4AT negative. Using the published sensitivity of the 4AT as 89.7% [21], we estimate this to be 25 of the 1141 who were 4AT negative. Therefore, results comparing outcomes between delirium and no delirium should be treated with caution. We suggest that given the well-described association of delirium to mortality, this is likely to have tempered our published results.

### What are the messages for routine clinical practice?

Specialist delirium teams have developed in hospitals in recent years with an aim to improve delirium management. Delirium teams improved the likelihood of delirium screening but actually reduced the likelihood of recognition. This contradiction could be due to inherent differences in how a delirium team diagnose delirium. Alternative solutions to improve routine delirium screening (e.g. embedded screening tools in admission documentation) were not associated with better screening rates. In the UK, 4AT screening in older trauma patients is financially driven; screening was similar to acute medicine, and yet recognition was reduced [42]. We consider that, although screening can improve recognition, wider training is needed alongside this. Trusts wishing to invest in delirium teams should ensure adequate training is provided to team members. We did not collect data on if formal delirium education or screening training had been targeted towards certain wards within individual hospitals. We were, therefore, unable to assess for intra-hospital factors that may lead to local variations within the same hospital, beyond specialty itself.

Possible delirium was defined as meeting individual but not all reference standard criteria. We consider this synonymous with subsyndromal delirium, defined as the presence of one or more symptoms of delirium, not meeting criteria for or progressing to delirium [43]. Standardised criteria to aid diagnosis do not exist; however, it is increasingly recognised as an important condition in its own right. Associations with increased length of stay [44], institutionalisation [45], and mortality [45] have been demonstrated. We report similar results. The majority of individuals with a positive 4AT score had either possible or definite delirium, and 4AT-positive status by itself predicts adverse outcomes. This highlights the value of the 4AT to identify those at high risk and reiterates that screening should become routine practice. We suggest that in an acute hospital environment, a positive 4AT highlights patients at increased risk of adverse outcome. We recommend pairing of a comprehensive delirium management strategy (such as the TIME Bundle from the Scottish Delirium Association) [46].

## Conclusions

Within the UK, delirium is highly prevalent amongst older hospital inpatients across specialties. Delirium is a severe condition associated with increased length of stay and mortality. Older adults with frailty are particularly vulnerable to delirium, and as frailty is associated with adverse outcomes in its own right, these patients exhibit greatest vulnerability. Unfortunately, our results suggest that delirium is less likely to be recognised in the frailest patients. We recommend that national quality improvement strategies should be implemented to increase screening and recognition of delirium, particularly focussing on patients with frailty and those admitted under surgical specialties.

## Supplementary information

**Additional file 1.** Proforma used for data collection including screening and reference standard diagnosis.

**Additional file 2.** Supplementary figures and tables as referenced within the main text.

**Additional file 3.** Strengthening the Reporting of Observational Studies in Epidemiology (STROBE) guideline checklist.

## Data Availability

The datasets used and/or analysed during the current study are available from the corresponding author on reasonable request.
