# Supplementary material for: Delirium is prevalent in older hospital inpatients and associated with adverse outcomes: results of a prospective multi-centre study on World Delirium Awareness Day
Source: BMC Med. 2019 Dec 14;17:229. doi: 10.1186/s12916-019-1458-7 (PMC6911703; doi:10.1186/s12916-019-1458-7)
Supplement: Supplementary file 1 — Additional file 1. Proforma used for data collection including screening and reference standard diagnosis. [file 12916_2019_1458_MOESM1_ESM.docx]

Patient ID (Unique hospital identifier): Age: Gender: M / F

| Dementia (notes) | Y / N / Probable | Delirium Screen (notes) | Y / N |
| --- | --- | --- | --- |
| Delirium diagnosis (notes) | Y / N | Date of admission |  |
| Specialty |  | Clinical Frailty Score (1-9; see reverse – use global assessment from screening and notes) |  |

**Part 1 Delirium Screening (~3 minutes)**

| **[1] ALERTNESS:** *Patients may be markedly drowsy (e.g. difficult to rouse, obviously sleepy during assessment) or agitated/hyperactive. If asleep, attempt to wake with speech or gentle touch on shoulder.*  Normal (fully alert, but not agitated, throughout assessment) **0**  Clearly abnormal **4**  Mild sleepiness for <10 seconds after waking, then normal **0** |
| --- |
| **[2] AMT4:** *Age, date of birth, place (name of the hospital), current year.*  No mistakes **0**  2 or more mistakes/untestable **2**  1 mistake **1** |
| **[3] ATTENTION:** *Ask the patient: “Please tell me the months of the year in backwards order, starting at December.” To assist initial understanding one prompt of “what is the month before December?” is permitted.*  Achieves 7 months or more correctly **0** Untestable (cannot start) **2**  Starts but scores <7 months / refuses to start **1** |
| **[4] ACUTE CHANGE OR FLUCTUATING COURSE:** S*ignificant change or fluctuation in: alertness, cognition, other mental function (e.g. paranoia, hallucinations) arising over the last 2 weeks and still evident in last 24hrs*  No **0**  Yes  **4**  4AT Total /12 |

**Only proceed to Part 2 assessment below if 4AT score > or = 4**

**Part 2 Delirium Assessment (DSM-5)**

1. Question patient: *Can you tell me what has been happening this morning? Can you tell me what this is? (show them a pen)*

2. Ask remainder of AMT10 below, check notes, speak to any staff/ carers immediately available.

Time (to nearest hour) Y/N Count backwards from 20-1 (all correct) Y/N
Monarch Y/N Recall dates of WW1 Y/N
Recognise two persons (e.g. doctor, nurse) Y/N Recall address (42 West Street) Y/N

| *Patients may show only minimal responses to verbal stimulation or be incapable of engaging with attempts at standardised testing or even interview. This inability to engage should be classified as severe inattention. Low-arousal states (of acute onset) should be recognised as indicating severe inattention and cognitive change, and hence delirium.* |
| --- |

3. mRASS score (see reverse): +4 +3 +2 +1 0 -1 -2 -3 -4 -5

| **a** | A disturbance in;  i) Attention- reduced ability to direct, focus, sustain, and shift attention FROM; *20-1, MOYB, observation* | **Yes** | No | ? |
| --- | --- | --- | --- | --- |
|  | ii) Awareness (reduced orientation to the environment) FROM; *mRASS not 0, observation* | **Yes** | No | ? |
| **b** | The disturbance;  i) Develops over a short period of time (usually hours to a few days) | **Yes** | No | ? |
|  | Ii) Represents a change from baseline attention & awareness and iii) tends to fluctuate in severity during the course of the day FROM: *History* | **Yes** | No | ? |
| **c** | An additional disturbance in cognition (e.g. memory deficit, disorientation, language, visuospatial ability, or perception). FROM; *Describe a pen, describe the morning, AMTS questions, observation* | **Yes** | No | ? |
| **d** | Exclusions- The disturbance in criteria A and C are;  i) Better explained by another pre-existing, established, or evolving neurocognitive disorder, or ii) Occur in the context of a severely reduced level of arousal such as coma. FROM; *mRASS not -4, -5* | Yes | **No** | ? |
| **e** | There is evidence from the history, physical examination or laboratory findings that the disturbance is a direct physiological consequence of another medical condition, substance intoxication or withdrawal, or exposure to a toxin, or is due to multiple aetiologies. FROM*; Notes (likely to be yes as in hospital)* | **Yes** | No |  |
|  |  | | | |
|  | **Probable Delirium Diagnosis – all items a,b,c and e ‘yes’, plus d ‘no’** | **Yes** | **No** |  |
|  | Possible delirium diagnosis – if any ‘?’ or e ‘no’ | Yes | No |  |

**REVISED DIAGNOSIS (After geriatrician review):** Delirium / Possible Delirium / No Delirium

**MOTOR SUBTYPE by DMSS-4 (see reverse):**


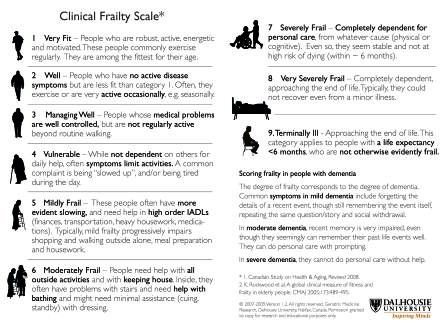


*
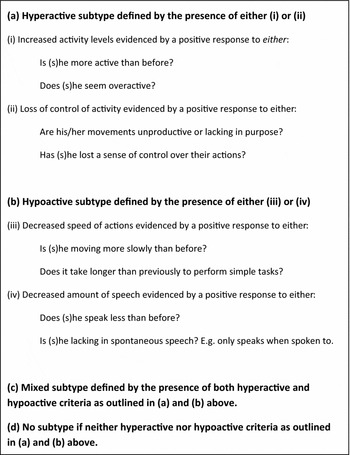
***DMSS-4**

**mRASS**


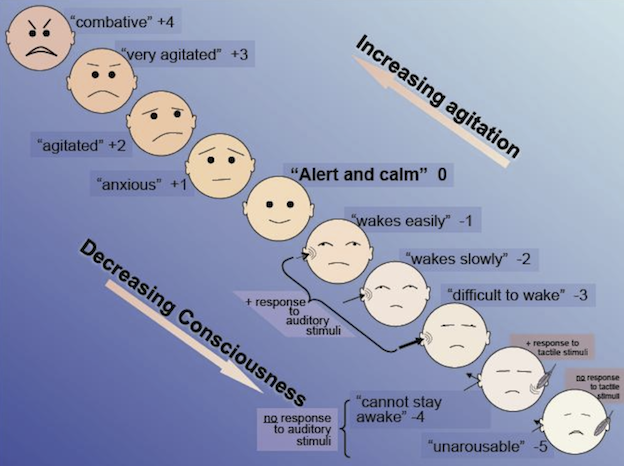


*Please use this space to make any general notes for discussion/ review with your local lead geriatrician:*
