# Supplementary material for: Delirium is prevalent in older hospital inpatients and associated with adverse outcomes: results of a prospective multi-centre study on World Delirium Awareness Day
Source: BMC Med. 2019 Dec 14;17:229. doi: 10.1186/s12916-019-1458-7 (PMC6911703; doi:10.1186/s12916-019-1458-7)
Supplement: Supplementary file 2 — Additional file 2. Supplementary figures and tables as referenced within the main text. [file 12916_2019_1458_MOESM2_ESM.docx]

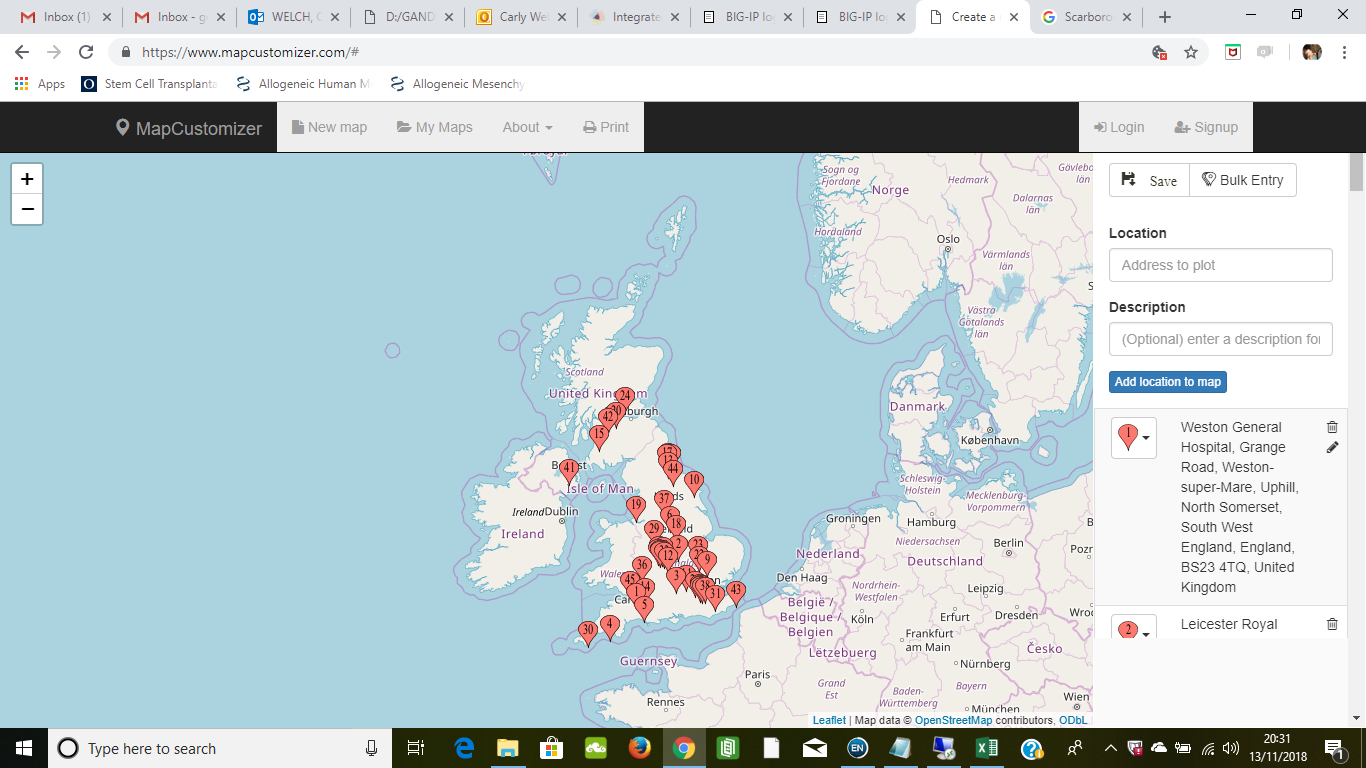


Figure S1 – Map of the sites that participated in this national study.

A total of 45 sites participated in this study. This map shows the locations of these sites across the UK.

|  | | All | No delirium, 4AT negative | 4AT positive, no delirium | Possible delirium | Delirium (DSM-5) | *P* |
| --- | --- | --- | --- | --- | --- | --- | --- |
| Age (mean, SD) | | 80.0 (8.3) | 78.9 (8.3) | 81.2 (8.0) | 82.3 (7.7) | 84.0 (7.4) | <0.001 |
| Gender | Female | 54.2% (798) | 52.9% (583) | 54.2% (32) | 52.2% (48) | 62.8% (135) | 0.102 |
| Dementia | No | 83.7% (1253) | 92.1% (1031) | 45.8% (27) | 54.1% (53) | 64.5% (142) | <0.001 |
|  | Probable | 3.0% (45) | 2.1% (23) | 6.8% (4) | 8.2% (8) | 4.5% (10) |  |
|  | Yes | 13.3% (199) | 5.9% (66) | 47.5% (28) | 37.8% (37) | 30.9% (68) |  |
| Specialty | Acute medicine | 43.0% (648) | 41.9% (473) | 4.92% (29) | 1.0% (40) | 47.8% (106) | <0.001 |
|  | Geriatric medicine | 17.6% (265) | 14.9% (168) | 25.4% (15) | 23.5% (23) | 26.6% (59) |  |
|  | Other medicine | 20.9% (315) | 23.0% (260) | 8.5% (5) | 19.4% (19) | 14.0% (31) |  |
|  | Stroke | 3.7% (56) | 3.5% (40) | 6.8% (4) | 8.2% (8) | 1.8% (4) |  |
|  | Other surgery | 2.5% (38) | 3.0% (34) | 1.7% (1) | 3.1% (3) | 0.0% (0) |  |
|  | General surgery | 6.0% (90) | 7.0% (79) | 3.4% (2) | 2.0% (2) | 3.2% (7) |  |
|  | Orthopaedic surgery | 6.3% (95) | 6.6% (74) | 5.1% (3) | 3.1% (3) | 6.8% (15) |  |
| CFS | 1 | 3.8% (56) | 4.9% (54) | 0.0% (0) | 1.1% (1) | 0.4% (1) | <0.001 |
|  | 2 | 10.3% (151) | 12.6% (140) | 0.0% (0) | 5.4% (5) | 2.8% (6) |  |
|  | 3 | 17.8% (261) | 21.7% (241) | 14.0% (8) | 4.3% (4) | 3.7% (8) |  |
|  | 4 | 17.5% (256) | 18.8% (209) | 12.3% (7) | 17.4% (16) | 11.1% (24) |  |
|  | 5 | 17.1% (251) | 16.7% (185) | 19.3% (11) | 8.7% (8) | 21.8% (47) |  |
|  | 6 | 19.7% (289) | 16.7% (185) | 26.3% (15) | 28.3% (26) | 29.2% (63) |  |
|  | 7 | 12.0% (176) | 7.0% (78) | 24.6% (14) | 29.3% (27) | 26.4% (57) |  |
|  | 8 | 1.5% (22) | 0.6% (7) | 1.8% (1) | 4.3% (4) | 4.6% (10) |  |
|  | 9 | 0.2% (3) | 0.1% (1) | 1.8% (1) | 1.1% (1) | 0.0% (0) |  |

Table S1 – Full demographics of patients separated according to all data collected

For the purposes of main data analysis, possible delirium was grouped with no delirium, and probable dementia was grouped with dementia. Due to small numbers, general and other surgery were combined for data analysis.

| Variable | Missing data % (N) |
| --- | --- |
| Age | 2.3% (35/1507) |
| Gender | 2.3% (35/1507) |
| Dementia status | 0.6% (10/1507) |
| Specialty | 0% (0/1507) |
| Clinical Frailty Scale | 2.8% (42/1507) |
| Subtype | 0.9% (2/222) |
| Screening performed by usual care team | 0.2% (3/1507) |
| Recognition by usual care team | 0% (0/222) |
| Length of stay or time to death | 3.6% (54/1507) |
| Mortality | 3.8% (58/1507) |
| Discharge documentation | 30.6% (68/222) of all patients with delirium  19.8% (38/192) of those alive at follow-up  7.8% (13/167) of those alive and discharged prior to follow-up; 25 still inpatients |

Table S2 – Rates of missing data for variables and outcome measures recorded during this study

Overall, rates of missing data were low for both variables and outcome measures. The exception to this is discharge documentation. However, the rates of missing data were lower after excluding patients who either died during admission or were still inpatients at the time of follow-up.

|  | Variables | | | Coefficient | SE | Wald | Freedom | *P* | OR | 95% Confidence interval for OR | |
| --- | --- | --- | --- | --- | --- | --- | --- | --- | --- | --- | --- |
|  |  |  |  |  |  |  |  |  |  | Lower | Upper |
| Patient factors | Age | | | **0.04** | **0.01** | **12.52** | **1** | **<0.001*** | **1.04** | **1.02** | **1.06** |
|  | Gender | | | 0.07 | 0.16 | 0.18 | 1 | 0.67 | 1.07 | 0.78 | 1.49 |
|  | CFS  (c.f. 1-3) |  | |  |  | **43.45** | **2** | **<0.001*** |  |  |  |
|  |  | 4-6 | | **1.57** | **0.31** | **26.25** | **1** | **<0.001*** | **4.80** | **2.63** | **8.74** |
|  |  | 7-9 | | **2.23** | **0.34** | **43.15** | **1** | **<0.001*** | **9.33** | **4.79** | **18.17** |
|  | Dementia | | | **0.67** | **0.18** | **13.16** | **1** | **<0.001*** | **1.95** | **1.36** | **2.79** |
| Disease-related | Specialty  (c.f. acute medicine) | |  |  |  | 9.34 | 5 | 0.096 |  |  |  |
|  |  |  | Geriatric medicine | -0.09 | 0.20 | 0.19 | 1 | 0.665 | 0.92 | 0.61 | 1.37 |
|  |  |  | Other medicine | -0.58 | 0.24 | 5.85 | 1 | 0.016 | 0.56 | 0.35 | 0.90 |
|  |  |  | Stroke | -0.91 | 0.63 | 2.11 | 1 | 0.146 | 0.40 | 0.12 | 1.38 |
|  |  |  | General and other surgery | -0.72 | 0.42 | 2.92 | 1 | 0.088 | 0.49 | 0.21 | 1.11 |
|  |  |  | Orthopaedic surgery | -0.05 | 0.34 | 0.02 | 1 | 0.893 | 0.96 | 0.49 | 1.86 |
| Hospital factors | Delirium team | | | 0.14 | 0.22 | 0.39 | 1 | 0.532 | 1.15 | 0.75 | 1.75 |
|  | Delirium guidelines | | | -0.13 | 0.25 | 0.26 | 1 | 0.607 | 0.88 | 0.54 | 1.44 |
|  | Delirium leaflet | | | -0.05 | 0.17 | 0.07 | 1 | 0.785 | 0.95 | 0.68 | 1.34 |
|  | Clerking tool | | | -0.30 | 0.21 | 2.14 | 1 | 0.144 | 0.74 | 0.49 | 1.11 |
|  | Geriatric medicine service in admissions unit | | | -0.12 | 0.17 | 0.45 | 1 | 0.501 | 0.89 | 0.64 | 1.25 |

Table S3 – Results of logistic regression analysis for the effect of variables upon the prevalence of delirium

Of those variables that were measured, increased age, increasing frailty status (both frail and very frail), and the presence of dementia were associated with increased odds of the presence of delirium. Specialty did not affect the prevalence of delirium after adjusting for other variables.

|  | Variables | | Coefficient | SE | Wald | Freedom | *P* | OR | 95% Confidence interval for OR | |
| --- | --- | --- | --- | --- | --- | --- | --- | --- | --- | --- |
|  |  |  |  |  |  |  |  |  | Lower | Upper |
| Hospital factors | Delirium team | | **0.71** | **0.16** | **19.40** | **1** | **<0.001*** | **2.03** | **1.48** | **2.80** |
|  | Delirium guidelines | | -0.36 | 0.19 | 3.43 | 1 | 0.064 | 0.70 | 0.48 | 1.02 |
|  | Delirium leaflet | | -0.25 | 0.13 | 3.47 | 1 | 0.063 | 0.78 | 0.60 | 1.01 |
|  | Clerking tool | | 0.13 | 0.17 | 0.57 | 1 | 0.449 | 1.14 | 0.81 | 1.60 |
|  | Geriatric medicine service in admissions unit | | -0.15 | 0.14 | 1.19 | 1 | 0.276 | 0.86 | 0.66 | 1.12 |
| Patient factors | Age | | **0.04** | **0.01** | **23.67** | **1** | **<0.001*** | **1.04** | **1.02** | **1.06** |
|  | Gender | | 0.14 | 0.13 | 1.26 | 1 | 0.261 | 1.16 | 0.90 | 1.49 |
|  | CFS  (c.f. 1-3) |  |  |  | 1.51 | 2 | 0.470 |  |  |  |
|  |  | 4-6 | 0.14 | 0.15 | 0.82 | 1 | 0.366 | 1.15 | 0.85 | 1.55 |
|  |  | 7-9 | -0.05 | 0.22 | 0.04 | 1 | 0.840 | 0.96 | 0.62 | 1.48 |
|  | Dementia | | 0.24 | 0.17 | 2.02 | 1 | 0.155 | 1.27 | 0.91 | 1.78 |
| Disease-related | Specialty  (c.f. acute medicine) |  |  |  | **12.25** | **5** | **0.032*** |  |  |  |
|  |  | Geriatric medicine | 0.10 | 0.17 | 0.37 | 1 | 0.545 | 1.11 | 0.79 | 1.55 |
|  |  | Other medicine | -0.17 | 0.17 | 0.94 | 1 | 0.331 | 0.85 | 0.60 | 1.19 |
|  |  | Stroke | -0.33 | 0.37 | 0.79 | 1 | 0.374 | 0.72 | 0.35 | 1.48 |
|  |  | General and other surgery | **-0.96** | **0.31** | **9.91** | **1** | **0.002*** | **0.38** | **0.21** | **0.70** |
|  |  | Orthopaedic surgery | 0.03 | 0.26 | 0.02 | 1 | 0.897 | 1.04 | 0.62 | 1.73 |

Table S3 – Results of logistic regression analysis for the effect of variables upon the likelihood of screening being performed by the usual care team

Of those variables that were measured, the presence of a local specialist delirium team and increased age were associated with increased odds of screening. Admission under general and other surgery was associated with reduced odds of screening when compared to acute medicine.

|  | Variables | | Coefficient | SE | Wald | Freedom | *P* | OR | 95% Confidence interval for OR | |
| --- | --- | --- | --- | --- | --- | --- | --- | --- | --- | --- |
|  |  |  |  |  |  |  |  |  | Lower | Upper |
|  | Screening | | **1.70** | **0.36** | **21.58** | **1** | **<0.001*** | **5.47** | **2.67** | **11.21** |
| Patient factors | Age | | 0.02 | 0.03 | 0.89 | 1 | 0.345 | 1.02 | 0.97 | 1.08 |
|  | Gender | | -0.17 | 0.36 | 0.21 | 1 | 0.646 | 0.85 | 0.42 | 1.72 |
|  | CFS  (c.f. 1-3) |  |  |  | **6.39** | **2** | **0.041*** |  |  |  |
|  |  | 4-6 | -1.02 | 0.72 | 2.03 | 1 | 0.155 | 0.36 | 0.09 | 1.47 |
|  |  | 7-9 | **-1.81** | **0.79** | **5.29** | **1** | **0.021*** | **0.16** | **0.04** | **0.77** |
|  | Dementia | | 0.47 | 0.39 | 1.50 | 1 | 0.221 | 1.61 | 0.75 | 3.42 |
| Disease-related | Specialty  (c.f. acute medicine) |  |  |  | **13.78** | **4** | **0.008*** |  |  |  |
|  |  | Geriatric medicine | 0.40 | 0.41 | 0.98 | 1 | 0.322 | 1.50 | 0.67 | 3.33 |
|  |  | Other medicine | -0.93 | 0.60 | 2.41 | 1 | 0.121 | 0.40 | 0.12 | 1.28 |
|  |  | Stroke | 1.03 | 1.37 | 0.56 | 1 | 0.455 | 2.79 | 0.19 | 40.99 |
|  |  | General, other, and orthopaedic surgery | **-3.18** | **1.10** | **8.39** | **1** | **0.004*** | **0.04** | **0.01** | **0.36** |
|  | Subtype (hypoactive) | | -0.34 | 0.35 | 0.93 | 1 | 0.334 | 0.71 | 0.36 | 1.42 |
| Hospital factors | Delirium team | | **-1.12** | **0.48** | **5.38** | **1** | **0.020*** | **0.33** | **0.23** | **0.84** |
|  | Delirium guidelines | | 0.28 | 0.61 | 0.21 | 1 | 0.646 | 1.32 | 0.40 | 4.33 |
|  | Delirium leaflet | | 0.26 | 0.38 | 0.45 | 1 | 0.505 | 1.29 | 0.61 | 2.73 |
|  | Clerking tool | | 0.74 | 0.48 | 2.36 | 1 | 0.125 | 2.10 | 0.81 | 5.41 |
|  | Geriatric medicine service in admissions unit | | 0.42 | 0.38 | 1.26 | 1 | 0.261 | 1.53 | 0.73 | 3.19 |

Table S5 – Results of logistic regression analysis for the effect of variables upon the likelihood of recognition of delirium by the usual care team

Screening for delirium was associated with increased odds of recognition, after adjusting for all other variables. Of those other variables that were measured, the presence of a local specialist delirium team, being very frail (CFS 7-9), and admission under general, other, or orthopaedic surgery were associated with reduced odds of recognition. Subtype did not affect the likelihood of recognition.

|  | Variables | | Coefficient | SE | Wald | Freedom | *P* | OR | 95% Confidence interval for OR | |
| --- | --- | --- | --- | --- | --- | --- | --- | --- | --- | --- |
|  |  |  |  |  |  |  |  |  | Lower | Upper |
| Patient factors | Age | | 0.03 | 0.03 | 1.16 | 1 | 0.282 | 1.03 | 0.98 | 1.09 |
|  | Gender | | -0.42 | 0.39 | 1.13 | 1 | 0.287 | 0.66 | 0.31 | 1.42 |
|  | CFS  (c.f. 1-3) |  |  |  | 0.39 | 2 | 0.823 |  |  |  |
|  |  | 4-6 | -0.17 | 0.79 | 0.04 | 1 | 0.835 | 0.848 | 0.18 | 4.00 |
|  |  | 7-9 | -0.41 | 0.86 | 0.23 | 1 | 0.631 | 0.66 | 0.12 | 3.55 |
|  | Dementia | | 0.52 | 0.41 | 1.60 | 1 | 0.206 | 1.68 | 0.75 | 3.72 |
| Disease-related | Specialty  (c.f. acute medicine) |  |  |  | 3.58 | 4 | 0.466 |  |  |  |
|  |  | Geriatric medicine | -0.07 | 0.49 | 0.02 | 1 | 0.883 | 0.93 | 0.36 | 2.43 |
|  |  | Stroke and other medicine | -0.53 | 0.69 | 0.58 | 1 | 0.445 | 0.59 | 0.15 | 2.27 |
|  |  | General and other surgery | -0.88 | 1.18 | 0.55 | 1 | 0.458 | 0.42 | 0.04 | 4.22 |
|  |  | Orthopaedic surgery | -1.83 | 1.11 | 2.74 | 1 | 0.98 | 0.16 | 0.02 | 1.40 |
| Hospital factors | Delirium team | | -0.23 | 0.52 | 0.20 | 1 | 0.657 | 0.79 | 0.28 | 2.21 |
|  | Delirium guidelines | | 0.25 | 0.81 | 0.10 | 1 | 0.758 | 1.28 | 0.26 | 6.22 |
|  | Delirium leaflet | | -0.40 | 0.44 | 0.84 | 1 | 0.359 | 0.67 | 0.29 | 1.58 |
|  | Clerking tool | | -0.09 | 0.53 | 0.03 | 1 | 0.861 | 0.91 | 0.32 | 2.58 |
|  | Geriatric medicine service in admissions unit | | 0.32 | 0.43 | 0.57 | 1 | 0.449 | 1.38 | 0.60 | 3.18 |

Table S6 – Results of logistic regression analysis for the effect of variables upon the likelihood of documentation of delirium on discharge summaries

No patient, disease-related, or hospital factors were predictive of documentation of delirium upon discharge summaries.

| Source | Type III Sum of squares | Freedom | Mean square | F | *P* | Partial Eta squared |
| --- | --- | --- | --- | --- | --- | --- |
| Corrected model | 7521.33 | 11 | 683.76 | 10.0 | <0.001 | 0.083 |
| Intercept | 109.03 | 1 | 109.03 | 1.60 | 0.207 | 0.001 |
| Delirium | **1524.13** | **1** | **1524.13** | **22.29** | **<0.001*** | **0.018** |
| Gender | 43.17 | 1 | 43.17 | 0.63 | 0.427 | 0.001 |
| Dementia | 104.11 | 1 | 104.11 | 1.52 | 0.217 | 0.001 |
| Specialty | **1360.34** | **5** | **272.07** | **3.98** | **0.001*** | **0.017** |
| CFS | **1524.27** | **2** | **762.13** | **11.15** | **<0.001*** | **0.018** |
| Age | **732.46** | **1** | **732.46** | **10.72** | **0.001*** | **0.009** |
| Error | 83263.87 | 1218 | 68.36 |  |  |  |
| Total | 204815.00 | 1230 |  |  |  |  |
| Corrected total | 90785.20 | 1229 |  |  |  |  |

Table S7 – Results of robust (bootstrapped) ANCOVA for the effect of delirium upon length of stay

Delirium was associated with increased length of stay in multivariable analysis. CFS, specialty, and age also additionally impacted upon length of stay.

| Source | Type III Sum of squares | Freedom | Mean square | F | *P* | Partial Eta squared |
| --- | --- | --- | --- | --- | --- | --- |
| Corrected model | 8008.74 | 13 | 616.06 | 9.05 | <0.001 | 0.088 |
| Intercept | 109.60 | 1 | 109.60 | 1.61 | 0.205 | 0.001 |
| Delirium | **2011.54** | **3** | **670.52** | **9.85** | **<0.001*** | **0.024** |
| Gender | 48.76 | 1 | 48.76 | 0.72 | 0.398 | 0.001 |
| Dementia | 282.33 | 1 | 282.33 | 4.15 | **0.042** | 0.003 |
| Specialty | **1395.65** | **5** | **279.13** | **4.10** | **0.001*** | **0.017** |
| CFS | **1300.43** | **2** | **650.22** | **9.55** | **<0.001*** | **0.015** |
| Age | **704.55** | **1** | **704.55** | **10.35** | **0.001*** | **0.008** |
| Error | 82776.46 | 1216 | 68.07 |  |  |  |
| Total | 204815.00 | 1230 |  |  |  |  |
| Corrected total | 90785.20 | 1229 |  |  |  |  |

Table S8 – Results of robust (bootstrapped) ANCOVA for the effect of delirium upon length of stay stratified by none, 4AT positive, possible, and definite delirium

Delirium, specialty, CFS, and age were all associated with differences in length of stay. Post-hoc tests were performed on all significant factors. The effect of delirium status upon length of stay has been presented in the main text.

| CFS (a) | CFS (b) | Mean Difference (a-b) | Bootstrap | | | | |
| --- | --- | --- | --- | --- | --- | --- | --- |
|  |  |  | Bias | SE | *P* | 95% Confidence Interval | |
|  |  |  |  |  |  | Lower | Upper |
| 1 | 2 | **-2.37** | **0.02** | **0.52** | **0.001*** | **-3.40** | **-1.32** |
|  | 3 | **-2.63** | **0.041** | **0.86** | **0.003*** | **-4.26** | **-0.88** |
| 2 | 3 | -0.50 | 0.01 | 0.83 | 0.750 | -1.41 | 1.95 |

Table S9 – Post-hoc tests of mean difference stratified by CFS

Increasing frailty status was associated with increased length of stay. There were statistically significant differences between fit and both frail and very frail patients; there was no difference between frail and very frail patients.

| Specialty (a) | Specialty (b) | Mean Difference (a-b) | Bootstrap | | | | |
| --- | --- | --- | --- | --- | --- | --- | --- |
|  |  |  | Bias | Std. Error | *P* | 95% Confidence Interval | |
|  |  |  |  |  |  | Lower | Upper |
| Acute medicine | Geriatric medicine | -0.79 | -0.17 | 0.70 | 0.251 | -2.20 | 0.55 |
|  | Other medicine | -0.97 | <0.01 | 0.63 | 0.127 | -2.24 | 0.29 |
|  | Stroke | -1.49 | -0.06 | 1.48 | 0.298 | -4.52 | 1.32 |
|  | General and other surgery | -0.05 | 0.02 | 0.75 | 0.946 | -1.60 | 1.43 |
|  | **Orthopaedic surgery** | **-4.24** | **0.03** | **1.13** | **0.001*** | **-6.41** | **-2.06** |
| Geriatric medicine | Other medicine | -0.19 | 0.018 | 0.80 | 0.806 | -1.72 | 1.42 |
|  | Stroke | -0.70 | -0.04 | 1.58 | 0.649 | -3.87 | 2.23 |
|  | General and other surgery | 0.73 | 0.04 | 0.90 | 0.404 | -0.93 | 2.56 |
|  | **Orthopaedic surgery** | **-3.45** | **0.05** | **1.25** | **0.003*** | **-5.90** | **-0.96** |
| Other medicine | Stroke | -0.52 | 0.06 | 1.51 | 0.741 | -3.58 | 2.24 |
|  | General and other surgery | 0.92 | 0.02 | 0.82 | 0.269 | -0.70 | 2.50 |
|  | **Orthopaedic surgery** | **-3.26** | **0.03** | **1.19** | **0.006*** | **-5.45** | **-0.93** |
| Stroke | General and other surgery | 1.44 | 0.08 | 1.55 | 0.350 | -4.64 | 1.52 |
|  | **Orthopaedic surgery** | **-4.19** | **0.01** | **1.25** | **0.001*** | **-6.73** | **-1.86** |
| General and other surgery | **Orthopaedic surgery** | **-4.19** | **-0.01** | **1.25** | **0.001*** | **-6.73** | **-1.86** |

Table S10 – Post-hoc tests of mean difference stratified by specialty

Patients admitted under orthopaedic surgery had increased length of stay compared to patients admitted under any other specialty. There were no other statistically significant differences in length of stay between specialties.

| Dementia status (a) | Dementia status (b) | Mean Difference (a-b) | Bootstrap | | | | |
| --- | --- | --- | --- | --- | --- | --- | --- |
|  |  |  | Bias | SE | *P* | 95% Confidence Interval | |
|  |  |  |  |  |  | Lower | Upper |
| No dementia | Dementia | 1.51 | -0.01 | 0.80 | 0.060 | -0.07 | 3.03 |

Table S11 – Post-hoc tests of mean difference stratified by dementia status

In post-hoc tests, dementia status did not have a significant effect upon length of stay.

| Source | Type III Sum of squares | Freedom | Mean square | F | *P* | Partial Eta squared |
| --- | --- | --- | --- | --- | --- | --- |
| Corrected model | 1088.68 | 11 | 98.97 | 1.00 | 0.450 | 0.67 |
| Intercept | 77.95 | 1 | 77.95 | 0.79 | 0.376 | <0.01 |
| Recognition | 41.62 | 1 | 41.62 | 0.42 | 0.518 | <0.01 |
| Gender | 85.28 | 1 | 85.28 | 0.86 | 0.355 | 0.01 |
| CFS | 138.60 | 2 | 69.30 | 0.70 | 0.498 | 0.01 |
| Dementia | 531.90 | 1 | 531.90 | 5.40 | 0.022 | 0.03 |
| Specialty | 272.25 | 5 | 54.45 | 0.55 | 0.738 | 0.18 |
| Age | 34.58 | 1 | 34.58 | 0.35 | 0.556 | <0.01 |
| Error | 15158.38 | 153 | 99.07 |  |  |  |
| Total | 46467.00 | 165 |  |  |  |  |
| Corrected total | 16727.07 | 164 |  |  |  |  |

Table S12 – Results of robust (bootstrapped) ANCOVA for the effect of recognition of delirium by the usual care team upon length of stay

Delirium recognition by the usual care team had no effect upon length of stay within this study. However, all delirium was recognised as part of this study due to its design within 48 hours of admission.

| Source | Type III Sum of squares | Freedom | Mean square | F | *P* | Partial Eta squared |
| --- | --- | --- | --- | --- | --- | --- |
| Corrected model | 1030.37 | 11 | 93.67 | 0.94 | 0.504 | 0.06 |
| Intercept | 77.30 | 1 | 77.30 | 0.78 | 0.380 | 0.05 |
| Subtype | 69.83 | 1 | 69.83 | 0.70 | 0.404 | 0.01 |
| Gender | 91.21 | 1 | 91.21 | 0.92 | 0.340 | 0.01 |
| CFS | 125.02 | 2 | 62.51 | 0.63 | 0.535 | 0.01 |
| Dementia | 431.11 | 1 | 431.11 | 4.33 | 0.039 | 0.03 |
| Specialty | 223.7 | 5 | 44.74 | 0.45 | 0.814 | 0.02 |
| Age | 34.28 | 1 | 34.28 | 0.34 | 0.558 | <0.01 |
| Error | 15047.00 | 151 | 99.65 |  |  |  |
| Total | 46422.00 | 163 |  |  |  |  |
| Corrected total | 16077.36 | 162 |  |  |  |  |

Table S13 – Results of robust (bootstrapped) ANCOVA for the effect of delirium subtype upon length of stay

Delirium subtype (hypoactive compared to others) had no impact on length of stay in this study.

|  | | Coefficient | SE | Wald | Freedom | *P* | OR | 95% Confidence interval for OR | |
| --- | --- | --- | --- | --- | --- | --- | --- | --- | --- |
|  |  |  |  |  |  |  |  | Lower | Upper |
| Delirium (unadjusted) |  |  |  | **25.45** | **3** | **<0.001*** |  |  |  |
|  | 4AT positive, no delirium | 0.54 | 0.54 | 0.98 | 3 | 0.323 | 1.71 | 0.59 | 4.93 |
|  | **Possible** | **0.93** | **0.38** | **5.85** | **1** | **0.016** | **2.53** | **1.19** | **5.36** |
|  | **Definite** | **1.23** | **0.25** | **23.71** | **1** | **<0.001*** | **3.44** | **2.09** | **5.65** |
| Delirium (adjusted) † |  |  |  | **14.37** | **3** | **0.002** |  |  |  |
|  | 4AT positive, no delirium | 0.53 | 0.57 | 0.88 | 1 | 0.342 | 1.70 | 0.56 | 5.15 |
|  | Possible | 0.77 | 0.41 | 3.45 | 1 | 0.063 | 2.15 | 0.96 | 4.82 |
|  | **Definite** | **1.07** | **0.29** | **13.97** | **1** | **<0.001*** | **2.90** | **1.66** | **5.08** |
| †Adjusted for age, gender, CFS, dementia status, and specialty | | | | | | | | | |

Table S14 – Results of logistic regression analysis for effect of delirium and possible delirium upon odds of mortality within 30 days

The presence of delirium was associated with increased odds of death within 30 days. The presence of possible delirium was associated with increased odds of death to 30 days before adjusting for other variables. In multivariable analysis, there was a non-significant statistical trend towards increased odds of death. 4AT was not predictive of risk of death to 30 days in those who had no evidence of delirium.

|  | Coefficient | SE | Wald | Freedom | *P* | OR | 95% Confidence interval for OR | |
| --- | --- | --- | --- | --- | --- | --- | --- | --- |
|  |  |  |  |  |  |  | Lower | Upper |
| 4AT positive (unadjusted) | **1.07** | **0.23** | **22.71** | **1** | **<0.001*** | **2.92** | **1.88** | **4.53** |
| 4AT positive (model 1) † | **0.93** | **0.26** | **12.85** | **1** | **<0.001*** | **2.55** | **1.53** | **4.24** |
| 4AT positive (model 2) ‡ | 0.68 | 0.36 | 3.64 | 1 | 0.057 | 1.99 | 0.98 | 4.03 |
| †Adjusted for age, gender, CFS, dementia status, and specialty  ‡Adjusted for age, gender, CFS, dementia status, specialty, and delirium status | | | | | | | | |

Table S15 – Results of logistic regression analysis for effect of 4AT positive status upon odds of mortality within 30 days

4AT positive status was associated with increased odds of death within 30 days after adjusting for age, gender, CFS, dementia status, and specialty. However, 4AT positive status was not associated with increased odds of mortality after adjusting for the presence of delirium.

|  | Coefficient | SE | Wald | Freedom | *P* | OR | 95% Confidence interval for OR | |
| --- | --- | --- | --- | --- | --- | --- | --- | --- |
|  |  |  |  |  |  |  | Lower | Upper |
| Recognition (unadjusted) | 0.35 | 0.41 | 0.73 | 1 | 0.392 | 1.43 | 0.63 | 3.21 |
| Recognition (adjusted) † | 0.46 | 0.45 | 1.06 | 1 | 0.304 | 1.59 | 0.66 | 3.87 |
| †Adjusted for age, gender, CFS, dementia status, and specialty | | | | | | | | |

Table S16 – Results of logistic regression analysis for effect of delirium recognition upon odds of mortality within 30 days

Delirium recognition by the usual care team had no effect upon 30 day mortality within this study. However, all delirium was recognised as part of this study due to its design within 48 hours of admission.

|  | Coefficient | SE | Wald | Freedom | *P* | OR | 95% Confidence interval for OR | |
| --- | --- | --- | --- | --- | --- | --- | --- | --- |
|  |  |  |  |  |  |  | Lower | Upper |
| Subtype (unadjusted) | 0.40 | 0.43 | 0.83 | 1 | 0.362 | 1.48 | 0.64 | 3.47 |
| Subtype (adjusted) † | 0.22 | 0.46 | 0.23 | 1 | 0.631 | 1.25 | 0.51 | 3.09 |
| †Adjusted for age, gender, CFS, dementia status, and specialty | | | | | | | | |

Table S17 – Results of logistic regression analysis for effect of delirium subtype upon odds of mortality within 30 days

Delirium subtype (hypoactive compared with all others) had no effect upon 30 day mortality within this study.
